# The Microscope—Its Misinterpretations

**Published:** 1877-10

**Authors:** 


### ARTICLE V.

### *The Microscope—Its Misinterpretations.*

"To enable the student to familiarize himself with the true power of the microscope, and to train his eyes to detect errors of vision, certain well-known test-objects are in general use, which are also convenient to test the quality and power of objectives. A favorite object of this class is the scale of the Podura, a minute insect, which dwells in remote nooks of dark and damp cellars, and similar localities.

This scale is usually mounted dry, and, when viewed under the compound microscope with suitable objectives, presents a surface studded with marks similar to the well-known note of exclamation (!)

This test-object has been for years the delight of microscopists possessing high powers, and a sharp definition of its peculiar markings as above mentioned was accepted as its true appearance and form.

For twenty-five years this scale was under constant examination by every grade of microscopists, from the grandees of the Royal Microscopical Society to the humble tyro, without any new or special feature being noticed, when on November 10, 1869, Dr. G. W. Royston Piggott, F. R. M. S., read a paper "On High-Power Definition" before the Royal Microscopical Society, and surprised the members by stating that all these years they had been gazing at the podura-scales, but had never yet seen its true markings. Dr. Piggott's paper described very fully what he had discovered as the true markings, and illustrated it with drawings which represented them to be distinctly of a beaded character; in fact, as dissimilar from the old accepted idea of their form as contrast could depict them.

Every microscopist was now hunting poduræ, and cellars damp and dismal were ransacked for the little scale-bearers, doubtless to the astonishment of numerous colonies of spiders, who must have been much provoked by this invasion, and thus commenced a controversy which is not yet concluded. Men equally eminent have taken opposite sides and expressed the most contrary opinions; and I now propose to give a brief *resume* of what has been said and done in regard to this subject, because the matter is full of instruction to those interested in microscopical research. Not that the markings of the podura are of the slightest importance or have any scientific significance, but the gravity of the conclusion which are sought hinges upon the fact that, if the views of Dr. Piggott are correct, our most eminent microscopists have been promulgating false and erroneous statements respecting the form of a well-known and common object; and, in whatever light the controversy is viewed, the humiliating confession must be made that they are still unable to determine the correct focus or the proper method of illuminating it.

Dr. Piggott commences by calling resolving the podura-scale "a difficult enterprise," and then describes the beaded appearance in the following manner: "Under a low power, as 80 or 100, the podura-scale is remarkable for its wavy markings, compared to watered silk; raising the power to 200 or 250, and using a side-light, the waviness disappears, and in its place longitudinal *ribbing* appears; with 1,200, they divide themselves into a string of longitudinal beads; but with 2,300 they appear to lie in the same plan and terminate abruptly on the basic membrane; in focusing for the beads attached to the lower side, the beadings appear in the intercostal spaces."

Respecting the old received views of the podura-scale, Dr. Piggott says: "With 300 to 500, the celebrated 'spines' appear, according to the size of the scale, as very dark tapering marks (like 'notes of admiration' without the dots ' ' '.) To see these clearly with 2,500 has been considered the *ne plus ultra* of microscopical triumphs, and it is consequently with no small diffidence that the writer ventures to traverse the belief of twenty-five years."

Thus was the gauntlet thrown down, and the challenge was at once accepted by various members of the Society, who, on the conclusion of the reading of the paper, at once, disputed the new doctrine. Mr. J. Beck was the first to express an opinion, and rather increased the confusion of the subject by stating that both the spines and the beads were illusory, and that the true structure of the podura-scale was a series of corrugations on one side, and that the reverse side was slightly undulating or nearly smooth, and that the notes of exclamation were due to refraction of light.

*December* 8, 1869.—The President, the Rev. J. B. Read, stated that he, with Dr. Miller and others, had interviewed Dr. Piggott, and was bound to say he had seen the beaded appearances, and it was clear to him, *now*, that in the best object-glasses small residuary aberration existed.

This slur upon the best object-glasses brought out Mr. Wenham with a paper, in the *Microscopical Journal* of June, 1870, in which he repudiated such error, and described the beaded appearance as an illusion, obtained by a trick of illumination, and by examining the scale with the microscope out of focus.

At the June meeting of the Royal Microscopical Society a letter was read from Colonel Woodward, of Washington, inclosing photographs of the podura-scale, showing what he considered to be the true appearance. These photographs showed the spines. Colonel Woodward, however, reserved his opinion, ond asked for a specimen of the true test podura-scale.

It would be tedious to continue the subject and give even an outline of the papers and discussions that have been provoked by this knotty question: I shall, therefore, conclude by stating that Colonel Woodward has since produced two photographs, showing the two aspects of the question; they are made from authentic scales, and are pronounced very perfect.

The fact that the most skillful microscopists of the age all differ upon the true appearances of a common and not very minute object, and the microscope itself presenting to the vision the most opposite appearances of one and the same object, should act as a caution to those who accept too readily theories based upon microscopical research; and suggests that, in the cause of justice, when life is at stake, single-handed evidence relating to the microscopical examination of apparent blood-stains should be verified at least by a second person before being accepted.

Thus we see that the so-called revelations of the microscope are but hieroglyphics, needing the interpretation of a mind of the highest culture, and that while the microscope is a good servant it is a bad master—mighty in the hands of a Huxley, but as useless to a man without the powers of discrimination as the chisel of Michael Angelo would be in the hands of a Modoc.—*Popular Science Monthly.*

